# 2-[4-(Chloro­meth­yl)phen­oxy]-4,6-dimeth­oxy­pyrimidine

**DOI:** 10.1107/S1600536812026220

**Published:** 2012-06-16

**Authors:** Shi-Hong Shen, Kui Hu, Li Sun, Xiao-Long Fan, Hong Dai

**Affiliations:** a Jiangsu Rugao Middle School, Rugao 226500, People’s Republic of China; b College of Chemistry and Chemical Engineering, Nantong University, Nantong 226019, People’s Republic of China; c College of Xinglin, Nantong University, Nantong 226019, People’s Republic of China

## Abstract

The title compound, C_13_H_13_ClN_2_O_3_, was synthesized in the course of the search for novel bioactive pyrimidine derivatives. The C—O—C angle at the phen­oxy O atom is widened to 119.87 (18)°. The dihedral angle between the pyrimidine and benzene rings is 64.2 (3)°.

## Related literature

For the biological activity of pyrimidine derivatives, see: Amin *et al.* (2011 ▶); Chen *et al.* (2009 ▶); Popova *et al.* (1999 ▶); Sagi *et al.* (2011 ▶); Stec *et al.* (2008 ▶). For related structures of 2-phen­oxy­pyrimidines, see: Shah Bakhtiar *et al.* (2009**a* ▶,b* ▶). For standard bond lengths, see: Allen *et al.* (1987 ▶).
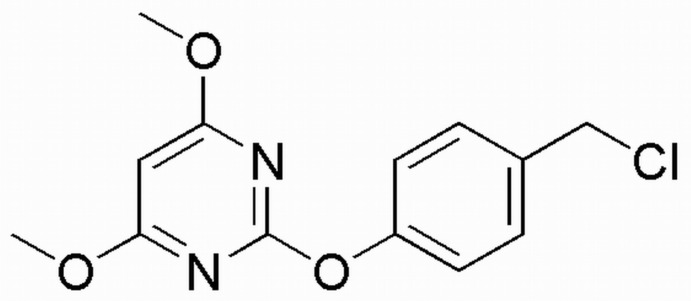


## Experimental

### 

#### Crystal data

C_13_H_13_ClN_2_O_3_
*M* *_r_* = 280.70
Monoclinic, 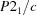

*a* = 8.3998 (17) Å
*b* = 23.145 (5) Å
*c* = 7.7967 (16) Å
β = 117.28 (3)°
*V* = 1347.2 (6) Å^3^
*Z* = 4
Mo *K*α radiation
μ = 0.29 mm^−1^
*T* = 113 K
0.30 × 0.25 × 0.20 mm

#### Data collection

Rigaku SCXmini diffractometer
Absorption correction: multi-scan (*CrystalClear*; Rigaku, 2008 ▶) *T*_min_ = 0.912, *T*_max_ = 0.942
11307 measured reflections
2358 independent reflections
1899 reflections with *I* > 2σ(*I*)
*R*_int_ = 0.039

#### Refinement

*R*[*F* ^2^ > 2σ(*F* ^2^)] = 0.054
*wR*(*F* ^2^) = 0.134
*S* = 1.09
2358 reflections
174 parameters
H-atom parameters constrained
Δρ_max_ = 0.62 e Å^−3^
Δρ_min_ = −0.52 e Å^−3^

### 

Data collection: *CrystalClear* (Rigaku, 2008 ▶); cell refinement: *CrystalClear*; data reduction: *CrystalClear*; program(s) used to solve structure: *SHELXS97* (Sheldrick, 2008 ▶); program(s) used to refine structure: *SHELXL97* (Sheldrick, 2008 ▶); molecular graphics: *SHELXTL* (Sheldrick, 2008 ▶); software used to prepare material for publication: *SHELXTL*.

## Supplementary Material

Crystal structure: contains datablock(s) global, I. DOI: 10.1107/S1600536812026220/yk2059sup1.cif

Structure factors: contains datablock(s) I. DOI: 10.1107/S1600536812026220/yk2059Isup2.hkl

Supplementary material file. DOI: 10.1107/S1600536812026220/yk2059Isup3.cml

Additional supplementary materials:  crystallographic information; 3D view; checkCIF report

## Figures and Tables

**Fig. 1.**
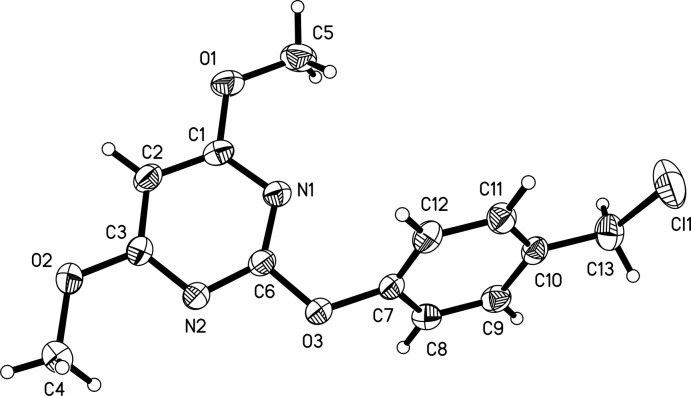
View of the title compound showing atom labelling scheme. Thermal ellipsoids drawn at the 30% probability level.

## supplementary crystallographic information

### Comment

In the past few years, many pyrimidine derivatives have drawn much attention in agrochemical and medicinal research because of their diverse bioactivities such as fungicidal, herbicidal and pharmacological activities (Popova *et al.*, 1999; Stec *et al.*, 2008; Chen *et al.*, 2009; Amin *et al.*, 2011; Sagi *et al.*, 2011). In the search of novel biologically active molecules, we have synthesized new pyrimidines. We report here the crystal structure of the target compound. It contains two planar groups, the benzene ring (C7/C8/C9/C10/C11/C12), and the substituted pyrimidine ring (N1/C1/C2/C3/N2/C6) (Fig.1). All bond lengths and angles in the title compound lie within normal ranges (Allen *et al.*, 1987) and are similar to those observed in the related 2-phenoxypyrimidines (Shah Bakhtiar *et al.*, 2009**a*,b*). The plane of pyrimidine ring makes a dihedral angle of 64.2 (3)° with the plane of benzene ring.

### Experimental

[4-(4,6-dimethoxypyrimidin-2-yloxy)phenyl]methanol (5 mmol) was dissolved in 50 ml of CH_2_Cl_2_. Thionyl chloride (5.5 mmol) was then added dropwise, while cooling on an ice bath. The resulting solution was heated to 298 K for 3 h, and the mixture was poured into 50 ml of ice water. The organic layer was washed with saturated brine(3 x 30 ml) and dried over anhydrous sodium sulfate. The solvent was evaporated under reduced pressure, and the residue was recrystallized from a mixture of petroleum ether/ethyl acetate to obtain colourless crystals. Mp: 354–356 K.

### Refinement

All H atoms were placed in calculated positions, with C—H = 0.93, 0.96 and 0.97Å for CH, CH_3_ and CH_2_ groups, respectively, and included in the final cycles of refinement using a riding model, with *U*_iso_(H) = 1.2*U*_eq_(C).

### Crystal data

**Table:**

C_13_H_13_ClN_2_O_3_	*F*(000) = 584
*M**_r_* = 280.70	*D*_x_ = 1.384 Mg m^−^^3^
Monoclinic, *P*2_1_/*c*	Mo *K*α radiation, λ = 0.71073 Å
Hall symbol: -P 2ybc	Cell parameters from 11126 reflections
*a* = 8.3998 (17) Å	θ = 3.1–27.6°
*b* = 23.145 (5) Å	µ = 0.29 mm^−^^1^
*c* = 7.7967 (16) Å	*T* = 113 K
β = 117.28 (3)°	Prism, colourless
*V* = 1347.2 (6) Å^3^	0.30 × 0.25 × 0.20 mm
*Z* = 4	

### Data collection

**Table:**

Rigaku SCXmini diffractometer	2358 independent reflections
Radiation source: fine-focus sealed tube	1899 reflections with *I* > 2σ(*I*)
Graphite monochromator	*R*_int_ = 0.039
Detector resolution: 14.22 pixels mm^-1^	θ_max_ = 25.0°, θ_min_ = 3.1°
ω and φ scans	*h* = −9→9
Absorption correction: multi-scan (*CrystalClear*; Rigaku, 2008)	*k* = −27→27
*T*_min_ = 0.912, *T*_max_ = 0.942	*l* = −9→9
11307 measured reflections	

### Refinement

**Table:**

Refinement on *F*^2^	Primary atom site location: structure-invariant direct methods
Least-squares matrix: full	Secondary atom site location: difference Fourier map
*R*[*F*^2^ > 2σ(*F*^2^)] = 0.054	Hydrogen site location: inferred from neighbouring sites
*wR*(*F*^2^) = 0.134	H-atom parameters constrained
*S* = 1.09	*w* = 1/[σ^2^(*F*_o_^2^) + (0.0495*P*)^2^ + 0.8134*P*] where *P* = (*F*_o_^2^ + 2*F*_c_^2^)/3
2358 reflections	(Δ/σ)_max_ < 0.001
174 parameters	Δρ_max_ = 0.62 e Å^−^^3^
0 restraints	Δρ_min_ = −0.52 e Å^−^^3^

### Special details

**Table:**

Geometry. All s.u.'s (except the s.u. in the dihedral angle between two l.s. planes) are estimated using the full covariance matrix. The cell s.u.'s are taken into account individually in the estimation of s.u.'s in distances, angles and torsion angles; correlations between s.u.'s in cell parameters are only used when they are defined by crystal symmetry. An approximate (isotropic) treatment of cell s.u.'s is used for estimating s.u.'s involving l.s. planes.
Refinement. Refinement of *F*^2^ against ALL reflections. The weighted *R*-factor *wR* and goodness of fit *S* are based on *F*^2^, conventional *R*-factors *R* are based on *F*, with *F* set to zero for negative *F*^2^. The threshold expression of *F*^2^ > σ(*F*^2^) is used only for calculating *R*-factors(gt) *etc*. and is not relevant to the choice of reflections for refinement. *R*-factors based on *F*^2^ are statistically about twice as large as those based on *F*, and *R*- factors based on ALL data will be even larger.

### Fractional atomic coordinates and isotropic or equivalent isotropic displacement parameters (Å^2^)

**Table:**

	*x*	*y*	*z*	*U*_iso_*/*U*_eq_	
C1	1.0641 (3)	0.96956 (10)	0.2361 (4)	0.0471 (6)	
C2	1.2474 (3)	0.97248 (11)	0.3080 (4)	0.0517 (6)	
H2	1.3138	1.0040	0.3785	0.062*	
C3	1.3259 (3)	0.92547 (10)	0.2683 (3)	0.0446 (6)	
C4	1.5903 (4)	0.87913 (13)	0.2908 (5)	0.0638 (8)	
H4A	1.5472	0.8434	0.3166	0.096*	
H4B	1.7177	0.8815	0.3689	0.096*	
H4C	1.5624	0.8807	0.1569	0.096*	
C5	0.7922 (4)	1.01267 (13)	0.2028 (5)	0.0692 (8)	
H5A	0.7339	0.9971	0.0744	0.104*	
H5B	0.7470	1.0507	0.2032	0.104*	
H5C	0.7689	0.9882	0.2882	0.104*	
C6	1.0600 (3)	0.88216 (10)	0.1124 (3)	0.0437 (6)	
C7	0.7881 (3)	0.82754 (10)	−0.0485 (4)	0.0454 (6)	
C8	0.7325 (3)	0.78267 (11)	0.0275 (4)	0.0524 (6)	
H8	0.8158	0.7593	0.1241	0.063*	
C9	0.5507 (4)	0.77293 (11)	−0.0426 (4)	0.0509 (6)	
H9	0.5123	0.7425	0.0072	0.061*	
C10	0.4246 (3)	0.80752 (11)	−0.1854 (3)	0.0448 (6)	
C11	0.4845 (3)	0.85228 (11)	−0.2603 (4)	0.0507 (6)	
H11	0.4017	0.8758	−0.3565	0.061*	
C12	0.6662 (4)	0.86220 (11)	−0.1929 (4)	0.0520 (6)	
H12	0.7054	0.8919	−0.2446	0.062*	
C13	0.2290 (4)	0.79504 (14)	−0.2462 (4)	0.0653 (8)	
H13A	0.2013	0.8076	−0.1442	0.078*	
H13B	0.2104	0.7536	−0.2602	0.078*	
Cl1	0.07844 (11)	0.82848 (5)	−0.46320 (16)	0.1037 (4)	
N1	0.9659 (3)	0.92397 (8)	0.1384 (3)	0.0452 (5)	
N2	1.2352 (2)	0.87930 (9)	0.1678 (3)	0.0450 (5)	
O1	0.9816 (3)	1.01557 (8)	0.2666 (3)	0.0647 (6)	
O2	1.5055 (2)	0.92689 (8)	0.3357 (3)	0.0565 (5)	
O3	0.9740 (2)	0.83341 (7)	0.0165 (3)	0.0576 (5)	

### Atomic displacement parameters (Å^2^)

**Table:**

	*U*^11^	*U*^22^	*U*^33^	*U*^12^	*U*^13^	*U*^23^
C1	0.0597 (15)	0.0372 (13)	0.0493 (14)	0.0004 (11)	0.0293 (13)	−0.0034 (11)
C2	0.0567 (15)	0.0407 (14)	0.0558 (15)	−0.0089 (11)	0.0242 (13)	−0.0105 (12)
C3	0.0450 (13)	0.0441 (14)	0.0448 (14)	−0.0059 (11)	0.0206 (11)	−0.0004 (11)
C4	0.0492 (15)	0.0636 (18)	0.079 (2)	0.0021 (13)	0.0293 (15)	−0.0057 (15)
C5	0.073 (2)	0.0577 (18)	0.091 (2)	0.0119 (15)	0.0504 (18)	−0.0029 (16)
C6	0.0476 (13)	0.0373 (13)	0.0476 (14)	−0.0038 (10)	0.0232 (11)	−0.0047 (10)
C7	0.0420 (12)	0.0390 (13)	0.0553 (15)	−0.0055 (10)	0.0223 (11)	−0.0138 (11)
C8	0.0535 (15)	0.0410 (14)	0.0590 (16)	0.0046 (11)	0.0227 (13)	0.0017 (12)
C9	0.0594 (15)	0.0390 (14)	0.0620 (16)	−0.0044 (11)	0.0345 (14)	0.0017 (12)
C10	0.0468 (13)	0.0457 (14)	0.0438 (13)	−0.0065 (11)	0.0224 (11)	−0.0096 (11)
C11	0.0515 (14)	0.0496 (15)	0.0443 (14)	−0.0016 (11)	0.0163 (12)	0.0027 (11)
C12	0.0577 (15)	0.0454 (15)	0.0546 (15)	−0.0115 (12)	0.0271 (13)	−0.0012 (12)
C13	0.0529 (16)	0.077 (2)	0.0670 (18)	−0.0118 (14)	0.0280 (14)	−0.0035 (16)
Cl1	0.0508 (5)	0.1230 (9)	0.1064 (8)	−0.0071 (5)	0.0095 (5)	0.0305 (6)
N1	0.0478 (11)	0.0396 (11)	0.0497 (12)	0.0002 (9)	0.0236 (10)	−0.0043 (9)
N2	0.0444 (11)	0.0408 (11)	0.0518 (12)	−0.0035 (9)	0.0238 (10)	−0.0049 (9)
O1	0.0698 (12)	0.0451 (11)	0.0882 (15)	0.0026 (9)	0.0442 (11)	−0.0150 (10)
O2	0.0443 (9)	0.0555 (11)	0.0666 (12)	−0.0082 (8)	0.0227 (9)	−0.0125 (9)
O3	0.0451 (10)	0.0445 (10)	0.0826 (13)	−0.0061 (8)	0.0288 (9)	−0.0208 (9)

### Geometric parameters (Å, º)

**Table:**

C1—N1	1.341 (3)	C6—O3	1.363 (3)
C1—O1	1.350 (3)	C7—C12	1.379 (4)
C1—C2	1.378 (4)	C7—C8	1.378 (4)
C2—C3	1.379 (3)	C7—O3	1.410 (3)
C2—H2	0.9300	C8—C9	1.385 (4)
C3—N2	1.337 (3)	C8—H8	0.9300
C3—O2	1.351 (3)	C9—C10	1.386 (4)
C4—O2	1.442 (3)	C9—H9	0.9300
C4—H4A	0.9600	C10—C11	1.391 (3)
C4—H4B	0.9600	C10—C13	1.514 (3)
C4—H4C	0.9600	C11—C12	1.387 (4)
C5—O1	1.435 (3)	C11—H11	0.9300
C5—H5A	0.9600	C12—H12	0.9300
C5—H5B	0.9600	C13—Cl1	1.760 (3)
C5—H5C	0.9600	C13—H13A	0.9700
C6—N1	1.322 (3)	C13—H13B	0.9700
C6—N2	1.332 (3)		
			
N1—C1—O1	119.3 (2)	C7—C8—C9	118.8 (2)
N1—C1—C2	123.4 (2)	C7—C8—H8	120.6
O1—C1—C2	117.2 (2)	C9—C8—H8	120.6
C1—C2—C3	115.5 (2)	C8—C9—C10	121.4 (2)
C1—C2—H2	122.3	C8—C9—H9	119.3
C3—C2—H2	122.3	C10—C9—H9	119.3
N2—C3—O2	118.8 (2)	C9—C10—C11	118.5 (2)
N2—C3—C2	124.0 (2)	C9—C10—C13	117.5 (2)
O2—C3—C2	117.2 (2)	C11—C10—C13	124.0 (2)
O2—C4—H4A	109.5	C12—C11—C10	120.7 (2)
O2—C4—H4B	109.5	C12—C11—H11	119.6
H4A—C4—H4B	109.5	C10—C11—H11	119.6
O2—C4—H4C	109.5	C7—C12—C11	119.3 (2)
H4A—C4—H4C	109.5	C7—C12—H12	120.4
H4B—C4—H4C	109.5	C11—C12—H12	120.4
O1—C5—H5A	109.5	C10—C13—Cl1	114.6 (2)
O1—C5—H5B	109.5	C10—C13—H13A	108.6
H5A—C5—H5B	109.5	Cl1—C13—H13A	108.6
O1—C5—H5C	109.5	C10—C13—H13B	108.6
H5A—C5—H5C	109.5	Cl1—C13—H13B	108.6
H5B—C5—H5C	109.5	H13A—C13—H13B	107.6
N1—C6—N2	129.5 (2)	C6—N1—C1	114.1 (2)
N1—C6—O3	119.1 (2)	C6—N2—C3	113.5 (2)
N2—C6—O3	111.5 (2)	C1—O1—C5	118.6 (2)
C12—C7—C8	121.2 (2)	C3—O2—C4	118.3 (2)
C12—C7—O3	121.5 (2)	C6—O3—C7	119.87 (18)
C8—C7—O3	117.1 (2)		
